# Prevalence of mental health problems among stranded international students during the COVID-19 pandemic

**DOI:** 10.1186/s40359-022-00917-2

**Published:** 2022-09-03

**Authors:** Shandana Iftikhar, Garon Perceval, Yining Fu, Chuan Zhou, Yongguo Cao

**Affiliations:** 1 School of Education, Soochow University, Room No. 5627, Building 1005, Dushu lake campus, Suzhou, China; 2 UQ Centre for Clinical Research, The University of Queensland, St Lucia, Australia

**Keywords:** COVID-19, Delta-variant, Risks factors of mental health, International students

## Abstract

**Background:**

The novel coronavirus disease (COVID-19) spread fast throughout China and the rest of the world, prompting the World Health Organization to declare a worldwide pandemic on March 11, 2020. Many countries have implemented travel bans, lockdowns, and stay-at-home policies to combat the spread of the COVID-19 pandemic. This study aimed to investigate the risk factors of mental health problems among international students stranded outside of China during the pandemic.

**Methods:**

A qualitative study was conducted among non-Chinese international students enrolled at Chinese universities who were stranded in their home countries. The participants were recruited using a purposive sampling technique. Following informed consent, in-depth interviews were conducted with the help of a semi-structured guide. Two independent investigators transcribed and coded the interview data. The investigators established themes after going through a detailed discussion.

**Results:**

Participants reported several mental health risk factors, such as a rise in hopelessness and level of uncertainty, worry, lost interest and focus, lack of support, unemployment and financial hardships, social pressure, behavioral and mood changes, sleep disorder, and increased smoking. These mental health problems will affect the concentration and deep learning, thereby increasing academic stress. In addition, we found that the outbreak of the delta-variant led to a further increase in these mental health risk factors.

**Conclusions:**

The pandemic scenario, along with international travel restrictions, increased the likelihood of mental health problems among stranded international students. Thus, preventing further rises in mental health disorders and reducing the effects of pandemic-related measures on stranded international students, such as researchers and policymakers can mitigate the pandemic's effects and achieve national or international health and educational goals. Adequate intervention for this group is strongly recommended.

## Introduction

The novel coronavirus disease (COVID-19) quickly spread around the world, prompting the (WHO) to announce a global pandemic on March 11, 2020 [1]. Governments have instituted local or international travel bans, lockdowns, and stay-at-home measures to combat the spread of COVID-19. Business and educational institutions have shut down, social engagements have been canceled, and non-essential staff has been told to work from home. Whereas these special precautions have successfully curbed the virus's transmission, additional problems are expected to emerge, including possible mental health crises in the aftermath of the COVID-19 Pandemic [2]. Increased psychological distress and worsened mental health symptoms have been recorded in earlier epidemics and traumatic global events [3]. Survey data from other hard-hit countries, like Italy, Spain, and Iran, have reported alarming rates of mental health symptoms [4]. COVID-19 is a traumatic event with worldwide implications; it is expected that increases in depression, anxiety, drug usage, and loneliness will be pervasive due to the pandemic's long-term effects of fear, social isolation, and economic troubles [5, 6]. Of particular interest is the impact of COVID-19 on university students' mental health, which has been studied in several nations, including Bangladesh, China, the United States, Australia, Canada, Jordan, and Germany. According to a study, 69.31% of university students in Bangladesh experienced mild to severe psychological effects as a result of the pandemic, as measured by the event-specific distress scale [7]. A Previous study has found that undergraduate students in China who were in their last year of university and had low sleep duration (less than 6 h per night) were more likely to suffer from Post-Traumatic Stress Disorder (PTSD) and depression symptoms [8]. Another study showed that 60% of students in the United States reported that the pandemic had a detrimental influence on their mental health, 90% of students were more concerned about their finances, 62% of students met the clinical cut-off for depression, and 47% met the clinical cut-off for generalized anxiety disorder [9]. Students from an Australian university reported poor well-being, with 31.1% having low well-being scores that may indicate clinical depression risk [10]. A meta-analysis of 136 investigations of COVID-19-affected populations indicated that at least 15–16% of the general population had anxiety or depressive symptoms [11]. According to a study conducted at a United States university, 48.14% of the non-probability sample had moderate-to-severe depression, while 38.48% had moderate-to-severe anxiety [12]. The majority of students, 71.26%, said their stress and anxiety levels had grown due to the epidemic, and fewer than half, 43.25%, said that they could adequately manage the stress [12]. Another study indicated that 54.5% of students in the United States exhibited anxiety or depressive symptoms [13]. In Jordan, mental health problems among university healthcare students were high [4]. Over 40% of students from Bavarian universities reported increased psychological pressure, whereas 17.3% claimed to have experienced reduced mental stress during the COVID-19 pandemic [14].

The rapidly spreading Coronavirus severely affected the educational system of China. The internationalization of higher education in China has made significant progress in recent decades, contributing to the transformation of China's education system into one of the world's largest and probably most productive systems. China is not only a major "sending" country, but it has also recently emerged as a significant "receiving" country in the global market for international education, attracting a growing number of students from all over the world [15]. In 2018, according to a report, 492,185 international students from 196 countries were enrolled in 1,004 higher education institutions in 31 provinces of China. Chinese government scholarships were awarded to 63,041 overseas students (12.81%), whereas 429,144 (87.19%) were self-funded (MOE, 2018). However, the COVID-19 pandemic has significantly impacted worldwide higher education, notably student mobility [16].

The United Nations Educational, Scientific, and Cultural Organization (UNESCO) have acknowledged that the global coronavirus pandemic impacts the educational system [17]. Many educational institutions worldwide have taken precautionary measures, such as shutting campuses or buildings, suspending classes, moving towards e-based teaching/learning curricula, exams, and deferring practicums [18]. Furthermore, many international students left China and returned to their home countries. Due to the virus's rapid spread, many countries imposed entrance restrictions on foreigners. As a result, many international students enrolled in Chinese universities were left stranded in their home countries with no clear timeframe for when they could return. However, many research studies examined students' mental health problems during the COVID-19 pandemic [17, 19]. Compared to domestic students, international students were more vulnerable to mental health problems during the pandemic [18, 20]. A study was conducted on international students of South Korean universities during the COVID-19 pandemic, which showed that the prevalence rates of sleep problems, anxiety, and depression among international students were 47.1%, 39.6%, and 49%, respectively [21]. Even under normal conditions, international students are more vulnerable to mental problems (e.g., depression), struggle with the local medical system, and are less inclined to seek psychological help [19, 22]. These studies showed that international students are more prone to mental health issues. Therefore, it is imperative to measure the impact of unexpected travel restrictions on international students' mental health.

Nonetheless, no work has been done on exploring the mental health status of stranded international students or international students stranded in their home countries due to pandemic-related travel measures. Therefore, the main objective of this study was to explore the mental health status of stranded international students. We looked into the mental health risk factors experienced by stranded international students from many countries. It is crucial to understand the impact of these factors in the global environment influenced by international travel restrictions.

## Materials and methods

### Study design and population

Qualitative research via semi-structured interviews was adopted for a detailed and in-depth understanding of stranded international students' mental health status. Qualitative research focuses on the nature, explanation, and comprehension of a phenomenon. The flexibility of semi-structured interviews enables the interviewer to pursue a sequence of less planned questions and investigate spontaneous issues highlighted by the interviewee [23]. An inductive thematic analysis was carried out [24]. The research was conducted using the COREQ (COnsoli-dated criteria for REporting Qualitative Research) checklist (Scheme S1) [25].

The targeted population selected for this study was international students of Chinese universities stranded in their home countries due to pandemic-related international travel restrictions. For the purposes of this study, stranded international students are defined as non-Chinese residents enrolled at Chinese universities but currently stranded outside of China and residing in their home countries.

### Development of data collection tool

To develop our semi-structured interview guide, we first consulted previous literature and various scales of mental health disorders to understand how mental symptomology has previously been operationalized. We consulted the following scales: The Center for Epidemiological Studies-Depression Scale [26], Depression-Anxiety-Stress-21 Scale [27]. General Anxiety Disorder-7 [28], the Patient Health Questionnaire- 9 [29], and the Perceived Stress Scale (PSS) [30]. Two researchers identified several indicators and incorporated them into our interview guides, such as the prevalence of anxiety symptomology (i.e., worry, fear), the prevalence of depression symptomology (i.e., loss of interest, hopelessness, negative thoughts), the prevalence of stress (i.e., academic, social, financial pressure), and prevalence of health issues due to stress (i.e., headache, fatigue).

### Participants

Our targeted population was non-Chinese resident international students enrolled at Chinese universities currently stranded outside of China due to pandemic-related control measures. Initial contact with approximately 3000 students was made via group messages placed on Facebook and WeChat pages for international student groups. We received responses from more than 1900 non-Chinese international students currently enrolled in Chinese universities. Due to time constraints, this study was delimited to the students currently on scholarships in China. Among these students, Chinese government scholarships (CSC) were awarded to 1052 students, while university scholarships were to 848 students (US). A total of 34 stranded international students, 22 male, and 12 female expressed interest in participating, and a time for the interview was set up. The degree status of some students' was continuous (n = 16), while others were suspended (n = 18). The participants were enrolled in masters (n = 23) and Ph.D. (n = 11) at Chinese universities, such as (Shenzhen University n = 2, Soochow university n = 7, Lanzhou university of technology n = 11, Dalian university of technology n = 10, Hebei normal university n = 1, Gansu agriculture university n = 1, Donghua university shanghai n = 1, Northwest normal university n = 1). The average age ranged from 22 to 35 years. The majority were from Pakistan (n = 12) and others from Vietnam (n = 1), Indonesia (n = 1), Nigeria (n = 4), Kashmir (n = 1), Uzbekistan (n = 1), Mauritania (n = 1), Yemen (n = 3), Sudan (n = 2), Tunisia (n = 1), Russia (n = 5), Palestine (n = 1), Magnolia (n = 1). For demographic details of each interviewee, see Table 1.

**Table 1:** Demographic details

Interview	Age	Gender	Nationality	Degree	Discipline	Degree status
1	27	Male	Pakistan	Masters	Chemical engineering	Suspended
2	25	Female	Pakistan	Masters	Material sciences	Continuous
3	31	Male	Indonesia	Ph.D.	Mechanical engineering	Suspended
4	29	Male	Yemen	Ph.D.	Business administration	Suspended
5	28	Female	Pakistan	Ph.D.	Mechanical engineering	Continuous
6	22	Male	Russia	Masters	Environmental sciences	Suspended
7	24	Female	Mongolia	Masters	Chemical engineering	Continuous
8	22	Female	Tunisia	Masters	Chemical engineering	Suspended
9	28	Male	Pakistan	Masters	Chemical engineering	Suspended
10	25	Male	Nigeria	Masters	Mechanical engineering	Suspended
11	26	Female	Nigeria	Masters	Applied sciences	Continuous
12	23	Male	Russia	Masters	Chinese culture	Suspended
13	29	Female	Nigeria	Masters	Pharmaceutics	Continuous
14	26	Female	Pakistan	Masters	Telecommunication	Continuous
15	27	Male	Yemen	Masters	Chemical engineering	Suspended
16	35	Female	Russia	Ph.D.	Social sciences	Suspended
17	29	Male	Pakistan	Masters	Mechanical engineering	Suspended
18	32	Male	Sudan	Ph.D.	Material sciences	Suspended
19	26	Male	Mauritania	Masters	Chemical engineering	Continuous
20	24	Male	Pakistan	Masters	Telecommunication	Suspended
21	25	Male	Pakistan	Masters	Material sciences	Continuous
22	31	Female	Uzbekistan	Ph.D.	Telecommunication	Suspended
23	23	Male	Kashmir	Masters	Mechanical engineering	Continuous
24	35	Female	Vietnam	Ph.D.	Material sciences	Continuous
25	24	Male	Russia	Masters	Chinese culture	Continuous
26	27	Male	Yemen	Masters	Mechanical engineering	Suspended
27	31	Female	Sudan	Ph.D.	Chemical engineering	Continuous
28	29	Male	Pakistan	Masters	Business administration	Suspended
29	26	Male	Pakistan	Masters	Pharmaceutics	Continuous
30	32	Male	Pakistan	Ph.D.	Chemical engineering	Suspended
31	30	Male	Nigeria	Ph.D.	Mechanical engineering	Continuous
32	26	Male	Palestine	Masters	Chemical engineering	Suspended
33	23	Female	Russia	Masters	Telecommunication	Continuous
34	28	Male	Pakistan	Ph.D.	Chemical engineering	Continuous

### Ethics

This research was approved by the university's ethics committee, where the first, second, and third authors were affiliated. All participants were assured that their personal information and identity would be kept anonymous and confidential. Participants signed an informed consent form and were told that participation in the study was entirely voluntary and that they could leave at any time. All methods were performed following the Declarations of Helsinki.

### Data collection

Prior to the interviews, all participants signed a written informed consent form. Participants were provided with verbal information about the study and could withdraw at any time during the interview. We conducted audio interviews with all the 34 participants via WeChat and WhatsApp following informed consent. Each interview lasted approximately 35 to 45 min. The interviewees were asked questions in English, Urdu, and Pashto languages. Twenty interviews were audio-recorded with consent, while the remainders were written down in a notebook.

The first author, a female Ph.D. student with prior experience conducting qualitative research interviews and primary education and training in qualitative methodology, conducted the interviews. The data reached its saturation level after conducting 25 interviews. We further conducted nine interviews due to the different background contexts of the participants but could not obtain any new information. Therefore, data collection was stopped after 34 interviews. This saturation level was deemed appropriate based on a review of prior research, which showed saturation levels being achieved within the first 12–24 interviews [31, 32].

The semi-structured interview guide included six predetermined questions in the current study. For example, "How long do you think you can go back and resume your studies? “Is your institution (in China) in touch with you?", "Do you have symptoms of any mental health problems/mental illness?", "Are you suffering from any anxiety due to the current situation of pandemic (i.e., feeling nervous, anxious, or cannot control worrying about things)?", "What gives you stress in the current situation?", "Do you drink or smoke?". Additional questions were asked according to the students' responses. All the interviews were translated to English, and the data was transcribed and compiled in a Word document.

### Data analysis

**Fig. 1:**
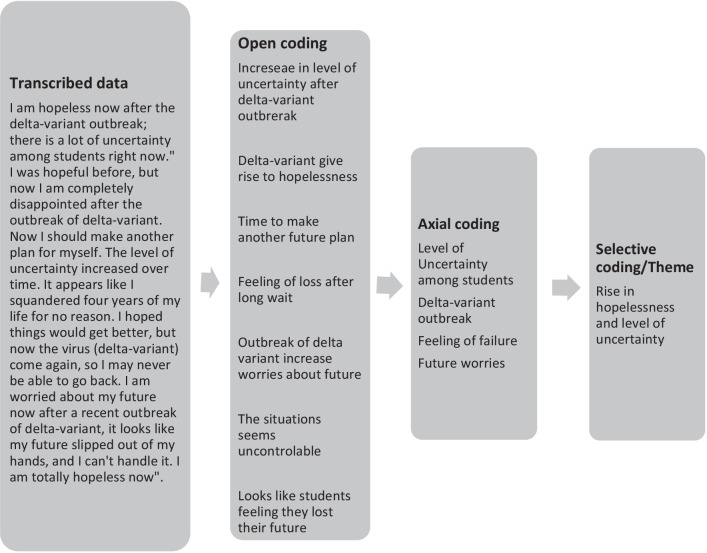
Illustration of coding process to the final theme

After receiving permission, all interviews were fully transcribed (written out line by line) with anonymized personal names and institution names. Grounded theory methodology was used to conduct an inductive content analysis [33]. Grounded theory is a method for systematically investigating personal experiences. This method entails discovering theory through data analysis, making it particularly well suited to investigating feelings, emotions, and experiences with no predetermined hypotheses to uncover new insights. We transcribed and compiled all the data in a Microsoft Word document for the current study and then disassembled the data. Disassembling data entails breaking it down and grouping it in meaningful ways. We carried out a coding process by identifying and highlighting sentences and phrases and labeling them with codes. Two team members employed a three-stage coding process comprising Open coding, Axial coding, and Selective coding using NVivo 12. In open coding, information was extracted from an MS Word document and organized into groups [34]. Meaningful data was highlighted, and initial codes were produced from the transcribed data. The same subject or idea information was grouped under the same heading. In contrast to open coding, Axial coding focuses on uncovering emergent themes and refines, aligns, and categorizes the concepts further [35]. This coding step is defined as the intermediary point of the coding process, covering the central concepts between open coding and final themes [34]. Selective coding is the final phase in qualitative methods, and it involves grouping all of the categories into one main category/theme, defining a single, cohesive theory [36]. Figure 1 depicts an example of the coding procedure.

The entire dataset was performed twice by two team members working independently and compared to ensure internal validity and avoid interpretation errors [31]. According to the guidelines established by Braun and Clarke, findings were then addressed within our interdisciplinary research team to guarantee agreement and ensure that key findings were not inadvertently overlooked [24]. Data were identified by assigning codes to the names of all the participants to ensure confidentiality. The first author searched for patterns in the data and grouped codes into possible themes after accurate coding of the entire dataset. To ensure reliability, the first, second, and third (corresponding) authors examined, revised, and refined these themes until they agreed on their content and labeling.

## Results

Our main finding was that the pandemic-related international travel restrictions adversely affected the mental health of many international students who went to their countries during the COVID-19 pandemic and have not yet returned to China. Most participants reported psychological and social factors associated with mental health symptomology or risk. Ten themes were identified: Rise in hopelessness and level of uncertainty, worry, lost interest and focus, lack of support, unemployment and financial hardships, social pressure, behavioral and mood changes, sleep disorder, increased smoking, and academic stress (Table 2). Some statements are presented as examples alongside participants' characteristics, and non-relevant sections for the quotes are indicated by *(……)*.

**Table 2:** Identified themes

Theme	Codes
A rise in hopelessness and level of uncertainty	Level of Uncertainty among students, Nanjing outbreak, Feeling of lost future, feeling of failure, future worries, hopelessness unpleasant feeling about returning back
Worry	Delay in educational goals, concerns about studies and future, unbearable situation, feeling depressed
Lost interest and focus	Feeling of laziness after a long break, enthusiasm begins to dwindle, difficulty in concentration
Lack of support	Institutional support, lack of information, negligence of authorities, no plan of action, lost hopes, feeling of unwanted students, suspension of monthly stipend
Unemployment and financial hardships	Hardships in finding jobs during the pandemic, lost jobs, less opportunities, job contracts, temporarily suspension of stipend, financial crisis
Social pressure	Questioning from friends and relatives, disconnecting social relationships
Behavioral and mood changes	Change in behavior, aggressive behavior, upset, mood changes
Sleep disorder	Difficulty in falling asleep, excessive sleepiness, disruption in sleep, active mind, dissatisfaction with online classes
Increased smoking	Help in reducing stress, feeling of helplessness, increase in smoking
Academic stress	Burden of publications, workload of experiments, and unavailability of resources at home countries, delay in graduation, online classes

### A rise in hopelessness and level of uncertainty

Participants reported that they were losing hopes of returning to China after the outbreak of delta-variant in Nanjing city, China. According to Chinese authorities, a commercial flight from Russia that landed in Nanjing on July 10 appeared to have initiated the outbreak by introducing the highly infectious delta-variant [37]. Before the outbreak of delta-variant, the participants had hopes that they could return soon. The sudden delta-variant outbreak has further tightened international travel restrictions, leading to rising hopelessness. The participants voiced their intention that now is the time to decide on their future and move on instead of further waiting.

"I am hopeless after the delta-variant outbreak; there is much uncertainty among students. (……). I was hopeful before, but now I am completely disappointed. (……)I think I should make another plan for myself". (Male, Ph.D. Indonesia, Interview 3).

Participants said that with the increased number of delta-variant cases, their level of uncertainty was also increased. The participants also stated that they were becoming more prone to negative thoughts. Their responses gave the impression that circumstances would probably never get better for them, and they would hardly be able to return. Negative future expectations might be described as hopelessness.

The level of uncertainty increased over time. It appears like I squandered four years of my life for no reason. I hoped before things would get better, but now the virus (delta-variant) has come again, so I may never be able to return. (……)I am worried about my future, it looks like my future slipped out of my hands, and I cannot handle it (……) feeling helpless now. (Female, Ph.D., Pakistan, Interview 5).

The participants were found disappointed and concerned with their future.

"I am distraught with this entire situation. It seems like I lost my future. I do not think this will end soon". (Female, MS, Nigeria, interview 11).

"You will find many more students, like me, who are disappointed. In the current situation, I do not think we will be able to return for the next two years".(Male, MS, Pakistan, Interview 21).

### Worry

Participants expressed their concerns regarding their education and future. They were worried about completing their studies on time due to being stranded. They believed they would be unable to achieve their goals, which would harm many of their other goals in the future.

"I am worried that my graduation may be prolonged. I wanted to finish my studies on time, move on, and build my future (……) as everything is interconnected, and if one plan fails, it affects all of your other plans and goals. ". (Female, Ph.D., Pakistan, Interview 5).

"We have been waiting a long time, and I believe that all of the students are worried and stressed in some way. Even if the situation improves, it will have a negative impact on our studies, future plans, and lives". (Male, MS, Russia, Interview 6).

Other factors contributing to participants' mental stress were family, financial, study concerns, and most importantly, concerns about the future.

"Of course, future worries are growing day by day. There is a total despondency (……). There are also a slew of other concerns, including finances, family, and obviously, our studies." (Male, Ph.D., Sudan, Interview 18).

"Everyone's mental state is in shambles. We have heard that new cases are on the rise in various countries (……). It appears like our studies are getting longer, which is becoming unbearable." (Male, MS, Pakistan, Interview 28).

### Lost interest and focus

The participants reported a loss of interest and focus due to the prolonged interruption to their studies. Furthermore, they stated that they were initially motivated to their studies, but their enthusiasm had waned since they were stranded in their countries due to the pandemic. Even if they return, concentrating on their studies will be tough for them, and it will take some time for them to do so.

"I was a very ambitious student. When I went to China, I was full of enthusiasm, ambitions, and a desire to do well. (……) Your enthusiasm starts to wane when there is such a long break." (Male, MS, Russia, Interview 6).

"Yes, it happens to me from time to time. I feel I am unable to concentrate on a single task. (……) We have had a long break. (……) It's becoming increasingly tough for us to concentrate on our schoolwork. (……) If we return, it will take time to refocus on our studies." (Female, MS, Pakistan, Interview 14).

### Lack of support

Participants voiced that the lack of institutional support increased their mental stress. They further added that if the universities had to develop a plan of action, it would have been easier for them to understand the situation, and they could have waited for a certain period. If the university gave them a satisfactory response, their anxiety would probably subside slightly.

I would not be so depressed if I knew what would happen next. The situation is very unpredictable. If the pandemic remains like this, what will be the strategy for international students? (……) Due to the lack of information from the university side, I have now given up hope and now looking for other opportunities. (Male, Ph.D., Yemen, Interview 4).

Participants said that, on the one hand, their studies were being hampered, and on the other hand, they were not receiving any meaningful response from their institution due to which they were suffering from mental distress. Moreover, the repeated contact of the participants with their institution for information also pointed out that there is a state of distress among them.

"I have to say with great regret that we have already lost about two years, and no one knows how many more years will be lost."

(Female, Ph.D., Russia, Interview 16).

I feel that no one cares about us (……) Our degrees are getting late, and our time is being wasted (……). If we only knew how long we would go, maybe we could do some work/job here (……). Whenever I ask my university about returning to China, they say we do not have any information yet, which is a pretty disappointing answer. (Male, MS, Yemen, Interview 26).

"The institutional lack of assistance irritates me the most. There is no appropriate information from the university side. They also discontinued our stipend, which is one of the key reasons for my stress.". (Male, MS, Pakistan, Interview 29).

### Unemployment and financial hardships

Participants reported having stable employment before moving to China to commence their studies. Participants found hardships in finding new jobs upon returning to their home countries during the pandemic.

"I was head of the department in a university when I got the scholarship; I quit my job and moved to China. I stayed there for only five months and came back due to the COVID-19 pandemic. (……) I did not know that this disease would become a pandemic and the borders would remain close for so long. (……) It is very challenging to get a job again in this circumstance (pandemic situation)." (Male, Ph.D., Yemen, Interview 4).

The whole world is in crisis right now. It is not easy to get a job in current circumstances. The participants associated their mental health with financial difficulties and unemployment. Furthermore, on returning these students to their homeland, their scholarship was suspended temporarily, which became a cause of suffering for them.

"The biggest challenge for me right now is my financial situation. It caused me mental distress. Due to the Pandemic, there are not enough opportunities to work. I work online now, but I do not get paid well". (Male, Ph.D., Sudan, Interview 18).

"Honestly, my current mental situation is very bad (…..). Before going to China, I used to teach in a college. I signed a contract with them to rejoin when I finished my studies. As long as I was getting the stipend, my financial condition was fine, but I have been in financial difficulties since our stipend was withheld. (…..) I am a single parent (mother) of a daughter. Can you imagine my mental state in this situation? Moreover, the pandemic situation in my country is dire. It is tough to find a job here". (Female, Ph.D., Vietnam, Interview 24).

There are some requirements for joining a job in the resident country of the participants. Candidates have to sign a contract with the institution, and they cannot leave that job until it is fulfilled. The pandemic is a disease we cannot predict how and when it will end. The participants gave us the impression that they were in the middle of nowhere. We found them in such a predicament that they could neither drop out of school nor choose a job.

"I have a family, so I need to earn money. Whenever I go for a job interview, they say to sign a contract for one year or more. However, I cannot go for such work because I am not sure when our university will call us back to China. What will I do with the contract if they call us back tomorrow? That is why I am doing some online work, but I am not getting enough money for the time being. If the universities continue to provide us with stipends, it will assist in alleviating at least our financial stress ". (Male, Ph.D., Pakistan, Interview 30).

### Social pressure

Participants said that they sometimes get tired of being asked questions repeatedly. They were going through a long study break; they got more upset when people asked questions over and over again.

"Yes, there is some social pressure, I think. When I meet my friends or relatives, they ask so many questions, for example, when will you go back? When will the borders open? Did you get any notifications? I am sick of hearing the same stuff repeatedly (……)". (Female, MS, Nigeria, Interview 11).

To avoid the questions of society, these participants tried not to meet the people. In that way, they did not have to face the people's questions. This action of participants indicates that they are moving towards loneliness.

"People around me, especially my relatives, keep asking about going back to China. These things bother me sometimes. I try to avoid them." (Male, MS, Yemen, Interview 26).

### Behavioral or mood changes

Participants stated behavioral or mood changes attributed to the international travel restrictions. After returning home, the students noticed mood changes. Due to the break in their education, they reported aggressiveness and irritability in their behavior.

"Yes, I feel a change in my behavior. I have never been shouted at my siblings before, but now I do it often. (…….) yes, you can say that my attitude is very aggressive now". (Female, MS, Tunisia, Interview 8).

"Yes, sometimes I get upset, but later I feel ok. (……) of course when you have nothing in your hands, and you cannot solve your problems, then it naturally causes irritability in your mood." (Male, Ph.D., Pakistan, Interview 30).

Participants with continuous degree status identified that the changes in their behavior were due to the online classes. The online classes were held in the morning according to Chinese time. They had to get up early, which disturbed their sleep. These students attributed the change in sleep patterns to their changing behavior.

"I had to get up in the middle of the night for online classes because of the time difference. (……)It had a bad effect on my daily routine, making me short-tempered. (……) I am not even satisfied with the online class". (Female, Ph.D., Sudan, Interview 27).

### Sleep disorder

Sleep disorders harm both the quantity and quality of sleep. [38]. Difficulty in falling asleep at night is a sign of insomnia or a strong desire to sleep excessively, referred to as hypersomnolence or hypersomnia [39]. Symptoms of sleep disruption were also found in our participants.

"Just a few months ago, I had insomnia. (……) I quickly fall prey to negative thoughts which in turn affect my sleep." (Female, MS, Tunisia, Interview 8).

"My sleeping pattern is getting worst. I often stay up till late at night and sleep throughout the day. (……) I cannot sleep well during the day. (……) I think my sleep duration is excessive". (Female, MS, Pakistan, Interview 14).

"I must sleep at least 12 to 14 h…………" (Male, MS, Mauritania, Interview 19).

Participants reported sleep disruption as a result of online classes. They identified that Sleep disorders and their associated mental illnesses are linked to changes in online class schedules and sleep–wake pattern.

"(……) I cannot sleep properly because of an inappropriate online class schedule. It keeps my mind active at night, making it difficult to sleep". (Male, MS, Pakistan, Interview 21).

### Increased smoking

Only male participants reported increased cigarette smoking rates. They smoke to reduce their stress. None of the female participants reported smoking or using any other substance.

"We are now helpless in these circumstances. (……) There is no other way for me to cope with my stress except to smoke". (Male, MS, Mauritania, Interview 19).

"I use cigarettes when I am upset or irritated. I attempt to calm myself down. Smoke makes me feel better" (Male, MS, Kashmir, Interview 23).

"Yes, I smoke more than before. (……)". (Male, Ph.D., Nigeria, Interview 31).

### Academic stress

Our study participants reported that the pressure of studies and research increased over time. Even if they wanted to continue their work in their country, the resources were not available. Lack of resources was the main reason for not completing the laboratory work or research work in their home country. They also stated that there were some degree criteria they were unable to meet, which resulted in a delay to their degree, such as publications. They suffered from mental stress due to the challenges in graduating and the lack of resources.

"My degree is about to complete, but the university said I have to publish an SCI paper to obtain the degree. Our country is not as developed as China. We do not have enough materials and chemicals for experiments. If I get the required material from somewhere, they demand much money (……) I am dealing with a lot of academic work. ". (Male, Ph.D., Indonesia, Interview 3).

"As time goes on, I am getting more and more anxious. My study has been continuous, but I will suspend it for the coming semester because it is impossible to complete the experimental work online. Studies-related stress is increasing day by day". (Female, Ph.D., Pakistan, Interview 5).

"I was supposed to graduate this year, but I could not finish my experiments (……). (Male, MS, Pakistan, Interview 17).

Participants indicated that online learning is somehow related to their mental stress; lack of interaction due to a poor internet connection and discussion in online classrooms could be the cause.

"Frequent internet disruptions result in poor interaction with the teacher, which makes me a bit stressful". (Male, MS, Mauritania, Interview 19).

"Yes, there is some academic pressure due to less understanding of lessons in the online lectures. (……) Many of the lessons in the online class are difficult to understand, and we are unable to have discussions with the teacher like we would in an offline class. (……)" (Male, MS, Pakistan, Interview 21).

## Discussion

To our knowledge, this is the first study to look into the mental health status of international students stranded outside of China due to pandemic-related international travel restrictions. Many international students from Chinese universities have gone to their home countries and have not returned to China yet (February, 2022). Therefore, we built this research on the urgent need to investigate the mental health status of stranded international students during the pandemic. The significant finding of the current study was that the international travel restrictions were adversely affecting our participants' mental health. None of the previous studies identified international travel restrictions causing several risk factors for mental health among stranded international students. These travel restrictions have led to a long break in studies of our population, due to which risk factors of mental health were found among them for example, rise in hopelessness and level of uncertainty, worry, lost interest and focus, lack of support, unemployment, and financial hardships, social pressure, behavioral and mood changes, sleep disorder, increased smoking, and academic pressure. The rapid spread of the delta-variant resulted in further increased local and international travel restrictions, which appeared to have dampened students' hopes of returning to China. Hopelessness was identified as a risk factor for mental health in a previous study; a high level of hopelessness in youngsters increases the risk of suicide, depression, and other psychopathologies [40]. Another study also associated hopelessness with mental health problems [41]. Our results further demonstrated that the participants suffered from disappointment and uncertainty due to the pandemic-related international travel restrictions, reflecting their concerns for the future. A previous study revealed that disappointment is made up of the fundamental emotions of grief and surprise; it might bring up feelings of sadness and surprise and ruin a person's mental health and well-being [42].

Moreover, our participants attributed their mental stress to unemployment and financial hardships. Consistent with our results, a study from Bangladesh found unemployment one of the key factors associated with poor mental health during pandemics [43]. Another prior study found that unemployment has a severe influence on the working-age population in Bosnia and Herzegovina, leading to poor mental health. The loss of a job has a predictive value [44]. The results of BaticMujanovic were similar to ours, but there was a population gap. The highest unemployment rate in the world was recorded at 44% in South Africa [45]. Karing [46] highlighted social and academic pressure, among other risk factors affecting students' mental health, similar to our findings. Portoghese highlighted high workload, attendance at courses, meeting deadlines, managing university and private life, and financial concerns as academic stressors. These stressors have been linked to an increased risk of distress and lower academic achievement [47]. In Geneva, Switzerland, a study looked at psychological well-being, distress, and related factors among undergraduate students pursuing eight different health-related tracks. They found that academic satisfaction was a stronger predictor of mental health than COVID-19, primarily used to predict stress and anxiety [48]. Furthermore, A recent study on a different population showed similar results, such as health authorities and the administration did not deliver accurate information, causing depression among the participants [49]. Additionally, according to another study on the prevalence of anxiety during the pandemic, 37.8% of participants reported worrying thoughts occasionally, while 23.8 percent said they had them all of the time [4], which is consistent with our findings. In terms of smoking, the majority of students 86.3%) (n = 201) did not smoke prior to the lockdown; however, 5.6% (n = 12) of the participants changed their smoking habits during the pandemic, with an equal number either quitting or starting to smoke [50]. They also indicated another factor consistent with our find. Almost all of the comments in the lifestyle and behaviors section indicated that the pandemic impacted their behavior, with more people stating that the pandemic harmed their behavior than a positive impact [50]. In the current study, few male participants reported an increase in smoking during the pandemic. Ting and Essau measure the addictive behavior among university students in Malaysia, they found in the past 30 days, 3.9% of students had smoked cigarettes which was very low was very low [51]. Previous studies, combined with the findings of our study confirmed that the factors we identified pose a risk to our population's mental health.

This study offers some advantages, including adequate data from individual interviews with stranded international students who have had indications of mental health problems due to international travel restrictions. The qualitative approach generated in-depth and comprehensive data on the phenomena investigated, particularly the mental health status of stranded international students and how risk factors adversely affect their mental health. This method allows the researcher to collect open-ended data, delve deeply into personal and often sensitive concerns, and study participant ideas, feelings, and opinions about a specific topic [52]. Unlike quantitative data, qualitative data is evaluated for in-depth meaning and process rather than being assessed in terms of frequencies and amount [53]. Participants were carefully recruited from various countries, schools, universities, and ages, resulting in a diverse and extensive data set. Recruitment continued until it was decided that the data information was rich and deep enough to successfully capture the study's aims of diversity and depth. As a result, data collection was completed when saturation was reached, i.e., when the researchers determined that sufficient rich and broad data had been obtained to address the research question [31, 32]. Data was initially independently coded by two authors, and the resulting themes were then thoroughly discussed by the research team, adding to the credibility of the data analysis [24]. The involvement of researchers with various backgrounds and expertise who systematically analyzed the data is also a strength. To increase the credibility of data, representative quotations from the transcribed texts were presented [54].

The interviews were limited to stranded international students of Chinese universities; we cannot generalize our findings to other countries. This research will be extended to other countries, providing rich literature for future research. Our findings add context to research identifying mental health risk factors during pandemics. Pandemic-related travel restriction measures were imposed to control the pandemic, but on the other hand, the stranded international students face other challenges due to these restrictions. Therefore, more work needs to be done on this topic, draw the attention of the institutions and policymakers, and develop suitable strategies and recommendations. Plans are needed for the safe return of students without increasing the risk of future waves of transmission. All the countries should pay special attention to the vaccination of their student that may allow them to go back and resume their studies in a usual way. The current findings highlight the need for mental health support services to be made available to promote these students' emotional and psychological well-being in their home countries. For example, telephone-based counseling services could be utilized to support international students [55]. Course-based online forums and internet-based counseling may also be viable options for increasing students' psychological well-being [56]. We urgently need research on evidence-based interventions to alleviate this distress.

## Conclusions

This study has shown a significant contribution to the literature. This is the first study to provide empirical evidence suggesting that many non-Chinese stranded international students enrolled in Chinese universities indicate symptoms of mental health problems. We identified several risk factors that influence the mental health of stranded international students, including a rise in hopelessness and level of uncertainty, worry, lost interest and focus, lack of support, unemployment and financial hardships, social pressure, behavioral and mood changes, sleep disorder, increased smoking, and academic stress. Given the prevalence of mental health issues among international students, mental health services for this population during the pandemic should include periodic mental health evaluations to ensure the early identification of students suffering from severe mental health problems. The pandemic scenario, along with international travel restrictions, increased the likelihood of mental health problems among stranded international students. Thus, preventing further rises in mental health disorders and reducing the effects of pandemic-related measures on stranded international students, such as researchers and policymakers, can mitigate the pandemic's effects and achieve national or international health and educational goals.

## Data Availability

Due to the confidentiality of participants' data, the datasets used in this study are not publicly available but are available from the corresponding author on reasonable request.
